# The students’ viewpoint on the quality gap in educational services

**Published:** 2014-07

**Authors:** MARZIYEH RAHIM KHANLI, HADI DANESHMANDI, ALIREZA CHOOBINEH

**Affiliations:** 1 Research Affairs office, Health school, Shiraz University of Medical Sciences, Shiraz, Iran;; 2 Department of Ergonomics, Health school, Shiraz University of Medical Sciences, Shiraz, Iran;; 3 Research Center for Health Sciences, Shiraz University of Medical Sciences, Shiraz, Iran

**Keywords:** Quality, Gap, Education, Questionnaire, Student

## Abstract

**Introduction:** Students and university community are social and human resources of the country. The students’ viewpoints about the quality of educational services can be considered as a basis for planning quality promotion and improving organizational performance. This study was conducted to determine the quality gap in educational services by the students of Health and Nutrition School of Shiraz University of Medical Sciences.

**Methods:** In this cross-sectional study, 140 students participated voluntarily (age range=19 to 40 years). The service quality (SERVQUAL) questionnaire was used for data collection. This questionnaire measured the quality gap in 5 dimensions of educational service including assurance, responsiveness, empathy, reliability, and tangibility. The students’ perception about the current conditions and their expectations as to optimal conditions can be determined, using this questionnaire. The score of the gap in quality of educational services is calculated from difference between perception and expectation scores. Due to non-normality of data, non-parametric tests were used. To this end, data were analyzed by statistical tests including Wilcoxon, Friedman, Kruskal-Wallis and Mann-Whiteny tests in SPSS 14.

**Results:** The results showed that there was quality gap in all 5 dimensions of educational services. The largest and the smallest gaps were observed in "responsiveness" with a mean±SD of -0.94±0.74 and in "reliability" with a mean±SD of -0.76±0.69, respectively. There was a significant difference in quality gap between the 5 dimensions (p<0.001).

**Conclusion:** According to the results, the students’ expectations were higher than their perceptions of current conditions; also, in all aspects of the services their expectations were not met. It is recommended that workshops on customer services, communication skills and personnel’s technical skills development should be planned and held. Also, allocating more resources for improving educational facilities and physical environment is recommended.

## Introduction

Students and university community are social and human resources of the country (1). As universities continue to become more student-oriented, student perceptions of higher educational facilities and services are becoming more important. Educational services quality, emphasizing student satisfaction, is a newly emerging field of concern in Iranian universities (2).

Berry (1995) suggests that services play important roles in enhancing the values and can positively influence organizational success. Understanding the customer expectations and performance is an essential component that can be used to enhance a company's service delivery (3).

“The SERVQUAL instrument (4)- widely recognized in the service sector as a multi-item scale developed to assess customer perceptions of service quality - has been used to assess service quality in higher education at the undergraduate level (5) as well as at the graduate and postgraduate levels (6)”.

This scale (SERVQUAL) assesses customers' perceptions and expectations of service quality along five dimensions: tangibles (the appearance of the school physical facilities, equipment, personal and communication materials), reliability (the school's ability to perform the promised services dependably and accurately), responsiveness (the school's willingness to help students and provide prompt service), assurance (the knowledge and courtesy of school office staff/faculty and their ability to convey trust and confidence), and empathy (the school office staff's and faculty's ability to provide a caring and individualized attention to students) (7).

Kebriaei and Roudbari (2005) conducted a study to determine the quality gap of educational services in Zahedan University of Medical Sciences. The results of this study showed that in all dimensions of service, quality gaps existed (1). Bradley carried out a study using the SERVQUAL questionnaire to determine the perceptions and expectations of Chinese students about the quality of educational services in postgraduate courses. It was found that in all five dimensions of service there were negative gaps (8).

Due to the above-mentioned points, the aim of the present study was to determine the quality gap of educational services in Health and Nutrition School of Shiraz University of Medical Sciences (SUMS). The results of this study can be considered as a basis to locate areas of performance where improvements are needed, or areas where resources may be utilized more efficiently.

## Methods

Service quality survey was conducted at the end of the winter semester in the academic year 2010/2011 in Health and Nutrition School of SUMS. Sample size was determined using the

$$n=\frac{z^{2}\times {\left(\text{SD}\right)}^{2}}{{\text{d}}^{2}}$$

formula in the 95% confidence level; 140 subjects participated in the study. It should be mentioned that we used the results of Kebriaei’s study in which the standard deviation (SD) of quality gap was 1.07 (7). First, using the proportion partition sampling method, in each field of study, the number of samples was determined and then the subjects were selected in systematic random method in these categories. All subjects voluntarily participated in the study after receiving oral information about the aims of the study. The SERVQUAL questionnaire was used as the data collection tool. Students were given verbal and written instructions and completed the questionnaire at the beginning of the session. The respondents remained totally anonymous. The questionnaire has five broad-based dimensions as judgment criteria including reliability, tangibility, responsibility, assurance and empathy (9). These dimensions are briefly defined below (10):

**Reliability:** Is the university reliable in providing the services? Does it provide as promised? Reliability reflects a university’s consistency and certainty in terms of performance. Reliability is the most important dimension for the consumer of services;

**Tangibility:** How are the service provider’s physical installations, equipment, people and communication materials? Since there is no physical element to be assessed in services, students often trust the tangible evidence that surrounds it when making their assessment;

**Responsibility:** Are university employees helpful and capable of providing fast services? It is responsible for measuring university and employees’ receptiveness towards students;

**Assurance:** Are employees well-informed, educated, competent and trustworthy? This dimension encompasses the university’s competence, courtesy and precision;

**Empathy:** This is the capacity a person has to experience another one’s feelings. This dimension measures how well educational staff behave toward the students and respects them.

The validity and reliability of this questionnaire has been reviewed by Arbouni et al. (11) in Zanjan University of Medical Sciences (Cronbach's alpha coefficient in expectation and perception sections were 0.95 and 0.94, respectively).

Students were asked to rate statements that would measure their expectations of the services provided by an ideal higher education organization. Then they were asked to rate another set of statements that would measure their perception of the actual services delivered to them in Health and Nutrition School of Shiraz University of Medical Sciences. The survey instrument (self-administered questionnaire) consisted of the following three sections:

- Demographic data (i.e. age, gender, year of study, etc.)
- Statements focusing on student expectations of higher education institutions in general
- Statements focusing on student perceptions of service quality

Instruction was given as to how to answer the questions on 5-point Likert scales. The scales were arranged so that “strongly agree” was coded as five, while “strongly disagree” was considered as score one. The participants were asked to circle the number that best matched their opinions for each question. In each dimension, the mean score of quality gap was determined. Then the mean of each dimension was compared with other dimensions.

Data were analyzed using statistical tests including Wilcoxon, Friedman, Kruskal-Wallis and Mann-Whiteny using SPSS 14 (SPSS Inc, Chicago, IL, USA). To determine the significant difference among the scores of perceptions, expectations and quality gap in each dimension with other dimensions, Wilcoxon test was used. Also, for determining the significant difference between the mean scores of the quality gap between five dimensions of quality, the Friedman test was used. Kruskal-Wallis analysis was used to determine the significant difference in mean scores of quality gap in different fields of study. Mann-Whiteny test was used to determine the significant difference in the mean scores of quality gap in different groups.

## Results

In this study, 72.1% and 27.9% of the subjects were females and males, respectively. Mean and standard deviation of the age of subjects was 22.92±4.27. Regarding the students' majors, 39% of the subjects were the students of occupational health, 18.2% environmental health, 16.1% public health, 10.6% epidemiology, 6.3% nutrition, 6.3% medical entomology, and 3.5% of them were health education and health promotion students.

Table 1 shows mean and standard deviation of the scores of perceptions, expectations and service quality gap as viewed by the students in various statements in each dimension.

**Table 1:** Mean and standard deviation of perceptions, expectations and quality gap related to educational services (n=140)

**Dimension of services**	**Perception**	**Expectation**	**Gap**
**Assurance**	**Mean±SD **	**Mean±SD**	
Facilitating discussion by teachers about the subject in the classroom	1.44±0.60	2.08±0.69	-0.64
Preparing the students for future jobs by providing theoretical and practical training in schools	1.47±0.69	2.74±0.97	-1.27
Allocating time outside of class hours by teachers to answer the students’ questions	1.60±0.74	2.37±0.96	-0.77
Providing sufficient resources to increase the students' awareness	1.44±0.62	2.44±0.86	-1.00
Empowering the teachers to have sufficient knowledge	1.30±0.51	1.88±0.65	-0.58
**Responsiveness**			
Accessibility to advisor and consultant whenever the students needs them	1.40±0.59	2.36±0.91	-0.96
Accessibility of students to management for transfer of their viewpoints and recommendations about educational issues	1.52±0.65	2.47±0.93	-0.65
Exertion of viewpoints and recommendations about educational issues in educational programs	1.50±0.74	2.59±0.92	-1.09
Provision of appropriate research resources to students for further studies	1.41±0.63	2.20±0.99	-0.79
Declaration of a timetable so that the students can refer to advisor for educational issues	1.55±0.77	2.47±0.94	-0.92
**Empathy**			
Application of relevant tasks and lessons	1.45±0.67	2.13±0.75	-0.68
Flexibility in satisfying the specific requirements of each student	1.50±0.69	2.35±0.88	-0.85
Suitability of the time of classes	1.53±0.79	2.69±0.91	-1.16
Existence of a quiet place for study in the school	1.40±0.63	2.71±0.68	-1.31
Appropriate behavior of educational staff with students	1.32±0.56	1.98±0.74	-0.66
The teachers’ respect as to the students	1.33±0.65	1.88±0.86	-0.48
**Reliability**			
Presentation of lesson subjects in each session at class in an organized manner	1.37±0.61	1.95±0.61	-0.58
Informing of students about his evaluation conducted	1.57±0.79	2.61±0.99	-1.04
Content provided in a manner that is understandable for students	1.46±0.68	2.20±0.71	-0.74
Consideration of better grades if the students attempt more	1.41±0.64	2.10±0.87	-0.69
Registration and maintenance of educational information of students without mistake	1.39±0.66	2.08±0.68	-0.69
Easy access to the research resources in the university	1.38±0.67	2.77±0.77	-0.79
Performance of activities by teachers and staff in due time	1.39±0.59	2.28±0.73	-0.89
**Tangibles**			
Decent appearance and professionalism of teachers and school staff	1.35±0.57	1.83±0.79	-0.48
Physical attractiveness (buildings, classrooms, chairs, etc.)	1.50±0.77	2.62±0.94	-1.12
Existence of modern equipment and materials (internet, library, overhead, etc.)	1.42±0.68	2.47±0.83	-1.05
Physical attractiveness of devices used in educational activities by teachers	1.47±0.73	2.43±0.84	-0.96

In Table 2, the mean and standard deviation of perception, expectation and gap scores of educational services in five dimensions are displayed. Statistical analysis revealed that the mean scores of students' perception and expectation (the gap between the current and expected condition) were significantly different in all 5 dimensions of services (p<0.001). In general, the mean of quality gap score of educational service between the current and expected condition in all 5 dimensions was negative. As shown, the smallest and the largest mean of quality gaps were related to reliability and responsiveness dimension, respectively.

**Table 2:** Mean and standard deviation of scores of perceptions, expectations and quality gaps of educational services in all 5 dimensions (n=140)

**Dimension of services**	**Perceptions**	**Expectations**	**Gap**	**p**
	**Med (25%-75%)**	**Med (25%-75%)**	**Med (25%-75%)**	
**Reliability**	1.14 (1-1.71)	2.14 (2-2.57)	-0.85 ((-0.14)-(-1.28))	<0.0001
**Tangibles**	1 (1-1.75)	2.25 (2-2.75)	-1 ((-0.25)-(-1.5))	<0.0001
**Assurance**	1.2 (1-1.8)	2.2 (2-2.6)	-1 ((-0.2)-(-1.4))	<0.0001
**Empathy**	1.16 (1-1.67)	2.33 (1.83-2.67)	-1 ((-0.16)-(-1.33))	<0.0001
**Responsiveness**	1.4 (1-1.8)	2.4 (2-2.8)	-1 ((-0.4)-(-1.4))	<0.0001
**Total quality **	1.32 (1-1.74)	2.32 (2.03-2.68)	-0.92 ((-0.23)-(-1.32))	<0.0001

* Wilcoxon test

Also, Friedman’s analysis revealed that the mean scores of quality gap in all 5 dimensions were significantly different (p<0.001). Wilcoxon analysis revealed that the mean score of quality gap among empathy and responsiveness, reliability and responsiveness and reliability with tangibility dimensions were significantly different (p<0.05).

Table 3 shows the frequency of quality gap in all 5 dimensions of educational services among the students. As seen, a high percentage of students reported that a negative gap existed in responsiveness dimension.

The results of this study showed that in the total quality of service, a small percentage of subjects believe that the quality gap was positive. 

**Table 3:** Frequency of the quality gap in 5 dimensions of educational services (n=140)

**Dimension of services**	**Positive gap**	**Lake of gap**	**Negative gap**
**Responsiveness**	10 (7.14)	15 (10.71)	115 (82.15)
**Empathy**	14 (10)	18 (12.85)	108 (77.15)
**Assurance**	12 (8.57)	21 (15)	107 (76.43)
**Reliability**	14 (10)	17 (12.13)	109 (77.87)
**Tangibles**	8 (5.71)	23 (16.42)	109 (77.87)
**Total quality **	11 (7.85)	62 (4.28)	123 (87.87)

In Table 4, mean and standard deviation of scores of quality gap in terms of the field of study, sex, entry year and educational status are presented. Kruskal-Wallis analysis revealed that the mean score of quality gap in different fields of study were significantly different (p<0.001).

Mann-Whitney test showed a significant difference between the mean score of quality gap among nutrition and medical entomology, environmental health, occupational health, public health and epidemiology. Also, this analysis showed a significant difference between the mean score of quality gap among medical entomology and public health, medical entomology and epidemiology, environmental health and occupational health and between occupational health and public health (p<0.05). This difference was not significant among the other fields of study (p>0.05).

**Table 4:** Mean and standard deviation of scores of quality gap in terms of field of study, sex, entry year and educational status (n=140)

**Variables**	**Score of gap** **Mean±SD**	**p**
**Field of study**		
Nutrition	-1.58±0.36	<0.001*
Medical entomology	-0.45±0.55	
Environmental health	-1.00±0.74	
Occupational health	-0.63±0.56	
Public health	-1.07±0.56	
Health education and health promotion	-0.90±0.8	
Epidemiology	-0.94±0.4	
**Sex**		
Female	-1.01±0.63	<0.001†
Male	-0.45±0.43	
**Year of entry**		
2010	-0.99±0.63	0.017†
2011	-0.76±0.64	
**Educational status**		
Main student‡	-0.89±0.63	0.016†
Transitional Student	-0.33±0.45	

* Kruskal-Wallis

† Mann-Whitney U‡ Those students who were accepted and started their education in SUMS from the early beginning of their education.

## Discussion

Generally, as the results of this study showed, there was a negative quality gap in all 5 dimensions of educational services. This indicates that students' expectations are beyond their perceptions. The results of Ruby's study showed that there were negative quality gaps in assurance, responsiveness, empathy, and reliability dimensions. But, in the tangibility dimension there was a positive quality gap. It means that the students' perceptions of the quality of educational services are beyond their expectations. Also, in Ruby's study, the most negative quality gap was in the reliability dimension, followed by the responsiveness and empathy dimensions, and the least negative quality gap was observed in the assurance dimension (12). The negative quality gaps in educational services show that opportunities exist for improvement of educational services.

The results of this study showed that the largest and the smallest gaps were related to "responsiveness" and "reliability". This result is in line with those of Aghamollaei et al.’s study (13). On the other hand, the results of a study in Kashan University of Medical Sciences (Bigdeli and Kebriaei) showed that the largest and the smallest quality gaps were in tangibility and reliability dimensions, respectively (1).

The largest quality gap in responsiveness dimension necessitates sensitivity and awareness of the students' demands, questions and complaints. The negative quality gap in this dimension and its statements indicates that supervisors and advisers are not accessible in case the students need them. Students don’t have easy access to administrators to express their viewpoints and suggestions regarding to the curriculum. Also, little attention is paid to introducing suitable references to students for further reading and the supervisors' consulting hours are not aptly and properly specified. Therefore, for improvement of the educational condition the quality gap should be minimized and more attention should be paid to the mentioned cases with customer-centered approach.

The smallest quality gap in reliability dimension represents that Health and Nutrition School is reliable in providing educational services. Also, this educational center has provided the quality services as promised and is consistent in terms of performance. 

The results of Kebriaei’s study in Zahedan University of Medical Sciences showed that quality gaps in assurance, responsiveness, empathy, reliability and tangibility dimensions were -1.54, -1.73, -1.55, -1.1 and -1.31, respectively. Additionally, the total quality gap was -1.49 (1). The result of the current study showed that level of quality gap was lower than that of Kebriaei’s study.

The negative quality gap in service dimensions can be used as a guideline for planning and allocation of resources (14). Thus, the five SERVQUAL dimensions can be classified into three priority groups for allocation of resources and organizational attempts to eliminate or reduce negative quality gaps, so that the responsiveness dimension is placed in the first priority, the tangibles, assurance and empathy dimensions are placed in the second priority, and the reliability dimension is placed in the third priority. If the aforementioned priorities are taken into account and the quality gap is attended to, the resulting improvement will benefit other dimensions as well; the negative quality gap (or quality improvements) in one dimension, the customers' viewpoint, can affect the negative quality gaps (or quality improvements) in other dimensions (15).

Due to the diversity of majors and educational levels in other universities with different facilities, equipment, staff and faculty members, the results of this study cannot be generalized to all. Hence, it is recommended that a similar study should be carried out in each university so that a model with more conformity is produced for planning to improve educational services quality.

This study was conducted in Health and Nutrition School of Shiraz University of Medical Sciences. Then the results of this study are not generalizable to other educational environments. 

The authors suggest that similar studies be performed in other educational contexts in Shiraz University of Medical Sciences in order to reach better conclusions about of educational services quality in this university.

## Conclusion

As the results of this study showed, quality gap of educational services in the current and expected conditions in all 5 dimensions was negative. Therefore, expectations of students were higher than their perceptions of the current conditions and their expectations were not satisfied. In this study, the largest mean of quality gap was related to responsiveness dimension and also a high percentage of students reported that a negative gap existed in responsiveness dimension. The largest quality gap existed in nutrition students’ viewpoints. Also, the mean quality gap was higher in female students, in those who were admitted in the university in 2010, and the students of the university rather than those who had transferred to our university as compared to other groups. 

“To survive in a highly competitive environment, higher education institutions (HEIs) are expected to raise the quality of services they provide” (16). “The key to improving services is to motivate employees because employees always perform the service. Clear service guidelines and knowledge of the school mission are necessary. Students always expect the staffs who deliver services to be formally dressed and guide them appropriately. Due to this fact, employees are always well groomed when proving services. We need to focus on the dimensions that do not show the delivery of better services and find out the area of improvement where services are weak” (17). Also, it is recommended that workshops be planned and held on customer services, communication skills and personnel’s technical skills development. Also, allocating more resources for improving educational facilities and physical environment is suggested.
